# A multidisciplinary critical appraisal and future research agenda for neuromodulation in spinal pain: beyond acute analgesia and correlative mechanisms

**DOI:** 10.1186/s12967-026-08528-w

**Published:** 2026-06-26

**Authors:** Dong’e Huang, Munan Lin, Junqing Dong

**Affiliations:** 1 Department of Traditional Chinese Medicine, 900th Hospital of PLA Joint Logistic Support Force, Fuzhou, China; 2 Department of Physiotherapy, 92435th Hospital of PLA, Ningde, 352103 China

We read with considerable interest the study by Wu et al. [1], which investigated repetitive peripheral magnetic stimulation (rPMS) for non-specific neck and low back pain. The authors’ innovative application of functional near-infrared spectroscopy (fNIRS) to probe central mechanisms represents a commendable and timely advancement in mechanistic pain research. However, a rigorous multidisciplinary appraisal reveals several critical methodological and interpretative limitations that must be addressed to solidify the conclusions and bridge the gap to meaningful clinical application.

## Sham control and blinding integrity: an unvalidated assumption

The use of a 90°tilt coil for sham stimulation, while operationally convenient, is a recognized methodological compromise. Superior approaches employ dedicated sham coils that replicate the full acoustic and crucially, the *somatosensory* experience (e.g., scalp paresthesia) of active treatment without delivering a suprathreshold electromagnetic field, a standard now expected in high-quality transcranial magnetic stimulation trials [2]. More fundamentally, this study did not assess blinding integrity. The absence of data on the participants’ or outcome assessors’ guesses regarding treatment allocation leaves the internal validity of this sham-controlled design uncertain. A significant treatment effect is less compelling if the possibility of effective unblinding cannot be excluded.

## Incomplete statistical reporting and modest correlative evidence

The authors presented significant between-group differences in post-intervention pain scores. However, for the fNIRS data—the core mechanistic finding—they reported only post-hoc between-group comparisons and within-group pre-post changes, failing to present the crucial *interaction p-values* from the stated two-way repeated-measures ANOVA (Time × Group). This omission obscures the statistical evidence for a differential *change over time* between the active and sham groups, which is central to claiming a specific intervention effect on the cortical activity. Furthermore, the reported correlations between changes in pain (∆NRS) and DLPFC activation (∆DLPFC), while statistically significant, were modest (*r* ≈ 0.24–0.31), accounting for less than 10% of the variance. This suggests that in this acute paradigm, DLPFC hemodynamics is a weak biomarker of the analgesic effect. The authors’ own citation [1, Ref 20] (Du et al.) also reported correlations without establishing causality, highlighting a field-wide reliance on associative rather than causal evidence.

## Acute design limits clinical meaning and contradicts acknowledged goals

The single-session, immediate post-treatment design, while useful for probing acute neurophysiological responses, severely constrains the study’s clinical message. Chronic non-specific spinal pain necessitates management strategies that provide sustained functional improvements. Demonstrating transient analgesia at 20 min offers minimal pragmatic guidance. We strongly concur with the authors’ own admission in their “Limitations” section: the single-session design is a “significant constraint,” and future work must investigate multiple sessions and the durability of the effect. The urgent clinical need, as underscored by recent guidelines [3], is for dose-response and multi-session randomized controlled trials (RCTs) measuring function, disability, and quality of life over weeks to months.

## Mechanistic overinterpretation: correlation is not causation

The concluding statement that rPMS works “by modulating DLPFC activity” constitutes an over-interpretation of the correlative data. The observed reduction in DLPFC activation could be an *epiphenomenon* or a *consequence* of reduced pain perception rather than its cause. To robustly demonstrate a central neuromodulatory mechanism, studies must move beyond correlation. Future work should integrate protocols such as conditioned pain modulation to assess endogenous inhibition or, more powerfully, employ interventional designs (e.g., using rTMS to inhibit or excite the DLPFC) to test whether directly altering DLPFC activity *causally* modulates pain perception in this context.

## Constructive pathway to definitive evidence

This study is a valuable hypothesis generator. To translate this promise into practice, a coordinated research agenda is required. First, future RCTs should employ validated active sham systems with formal blinding checks. Second, pragmatic, multi-session trials comparing optimized rPMS parameters against standard care with pre-registered functional endpoints must be prioritized. Third, to truly dissect the mechanisms, multimodal assessments (e.g., combined fNIRS-EEG for neurovascular coupling or quantitative sensory testing) are essential to disentangle peripheral from central effects. Finally, as suggested by the authors’ findings, causal interventional studies targeting the DLPFC are the logical next step to validate its role.

In summary, Wu et al. provided intriguing preliminary evidence linking rPMS-induced analgesia to DLPFC activity. However, the methodological limitations concerning sham control, statistical reporting, and the inherently limited clinical relevance of an acute design, coupled with the acknowledged need for longer-term studies, mean that the mechanistic claims remain provisional and the clinical utility unproven. We encourage authors and the research community to build upon this foundation with more rigorous, clinically oriented, and causally informative studies that the field of neuromodulation for chronic pain urgently requires.

## Data Availability

Not applicable. (No new datasets were generated or analyzed for this commentary. The letter critiques a publicly available study).
